# Genistein protects against ultraviolet B–induced wrinkling and photoinflammation in in vitro and in vivo models

**DOI:** 10.1186/s12263-022-00706-x

**Published:** 2022-02-24

**Authors:** Sheau-Chung Tang, Yu-Ping Hsiao, Jiunn-Liang Ko

**Affiliations:** 1 Department of Nursing, National Taichung University of Science and Technology, Taichung, 403 Taiwan; 2 Institute of Medicine, Chung Shan Medical University, No.110, Sec. 1, Chien-Kuo N. Road, Taichung, 402 Taiwan; 3 Department of Dermatology, Chung Shan Medical University Hospital, No.110, Sec. 1, Chien-Kuo N. Road, Taichung, 402 Taiwan; 4 Department of Medical Oncology and Chest Medicine, Chung Shan Medical University Hospital, Taichung, 402 Taiwan

**Keywords:** Genistein, Ultraviolet B, Skin, Inflammation, Photoaging

## Abstract

**Background:**

Chronic exposure to ultraviolet (UV) rays causes severe skin damage by inducing oxidative stress and inflammation. Identifying a safe and natural substance for skin protection is a crucial research goal.

**Objective:**

The aim of this study was to clarify the effects of genistein on skin inflammation and photoaging by using 3 models (humans: skin parameters; animals: wrinkle formation; and cells: anti-inflammatory effects).

**Methods:**

Food frequency questionnaire data and serum and skin parameter data from 120 volunteers (a group with a genistein-rich diet [RG group] and a control group). Human keratinocytes were pretreated with genistein before ultraviolet B (UVB) irradiation. Genistein was topically applied to the dorsal skin of rats.

**Results:**

The blood samples of the RG group had lower serum uric acid levels and blood urea nitrogen levels. The dynamic elasticity level in the RG group was higher than that in the controls. Genistein pretreatment suppressed the expression of proinflammatory cytokines (CXCL1, IL-1, MIF, and PLANH1) and the proteins released by UVB-treated keratinocytes. Topical application of genistein to the dorsal skin of rats reduced the severity of UVB-induced wrinkling. Both intake and topical application of genistein combated UVB-induced inflammation and aging.

**Conclusions:**

Genistein could be used as a safe and natural compound for use in novel anti-inflammatory agents for topical application.

**Graphical abstract:**

The experimental design procedure, including the skin parameter and blood serum measurements of 137 participants. Genistein-rich compounds provide protection against UVB-induced inflammation, as determined using in vitro and in vivo animal model experiments. 
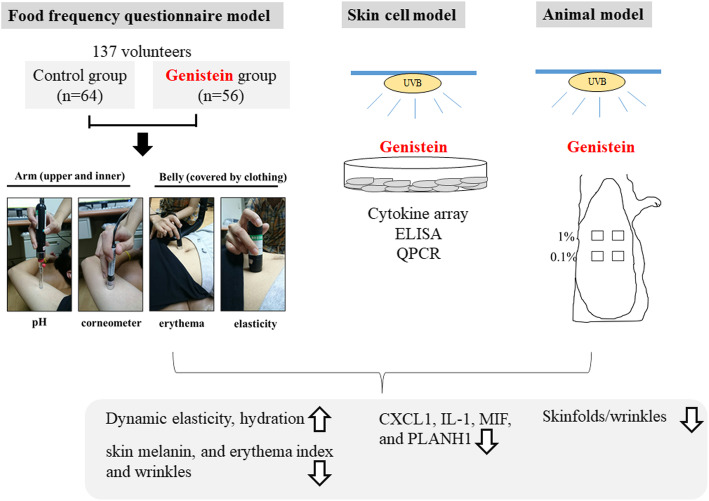

**Supplementary Information:**

The online version contains supplementary material available at 10.1186/s12263-022-00706-x.

## Highlights:

- Genistein suppressed UVB-induced proinflammatory cytokines (CXCL1, IL-1, MIF, and PLANH1) gene and protein levels in skin cells.
- Genistein reduced the skin folds and wrinkles induced by UVB in the animal model.
- Positive correlation between the RG diet and skin hydration.

## Background

UVB is a known common environmental carcinogen that causes skin aging through the production of reactive oxygen species (ROS) [1–3]. Latreille et al. revealed that intake of polyunsaturated fatty acids, such as docosahexaenoic acid and eicosapentaenoic acid, had an inverse association with UVB-induced photoaging [4]. Dietary consumption of aloe sterols was reported to protect mouse skin against chronic UVB damage [5]. These results indicate that foods can prevent skin aging.

The isoflavone genistein (5,7,40-trihydroxyisoflavone) is abundant in vegetable protein sources, such as soybean. Epidemiological and experimental studies have indicated that genistein may prevent certain symptoms and conditions, such as menopausal symptoms, skin cancer, heart disease, and melanoma [6–8]. Genistein decreased serum alanine transaminase and aspartate transaminase levels in a model of rat liver cancer [9]. Furthermore, it blocked apoptotic and necrotic pathways by modulating the expression of proapoptotic genes and proteins [10]. Evidently, genistein has multiple biological functions.

Certain foods and beverages, such as green tea, fish oil, and soybean, possess anti-inflammatory and antioxidative properties, which can help prevent or alleviate various conditions, such as hypertension, gout, and allergies [11–13]. Intake of plant protein is a very healthy way of eating. A diet rich in genistein (RG diet) protects against lower-abdominal obesity, inflammation, and insulin resistance [14]. However, whether genistein-rich food additionally affects physical appearance or can prevent skin disease is unknown.

No evidence is available on the anti-inflammatory and antiphotoaging effects of long-term genistein supplementation on UVB-treated human skin models. Therefore, we hypothesized that exposure to genistein would protect against UVB-induced wrinkle formation and photoinflammation in in vitro and in vivo models. In this study, we sought to understand (1) the differences between the physiological activity of human skin in the RG group and the control group, (2) the action of genistein against cell inflammation caused by UVB light exposure, and (3) how genistein can be applied to induce antiwrinkle effects in animals. We administered the food frequency questionnaire (FFQ) to 120 healthy volunteers and analyzed their genistein intake data, serum, whole blood, and urine samples. A cytokine array and animal model were used to determine the mechanism of genistein’s protective effects against UVB-induced photoinflammation.

## Results

### Genistein reduces blood urea nitrogen level

The age range of the 120 participants was 54–56 years. In the RG (*n* = 56) and control groups (*n* = 64), 68% and 56% of the participants were women, respectively. On average, the participants in the RG group had a larger waist than those in the control group, but age and body mass index (BMI) did not differ significantly between the 2 groups (Table 1). No significant intergroup differences were noted in terms of blood electrolytes, such as iron, potassium, sodium, calcium, and chloride (Table 2).

**Table 1:** Participants’ basic demographic characteristics

Groups	Control (***N*** = 64)	RG(***N*** = 56)
**Mean age in years (± SD)**	54.04(± 12.30)	56.35 (± 11.55)
**Female**	56%	67%
**Average waist (inches)**	29.3(23.2~37.8)	33.08(24.5~42.0)
**BMI (± SD)**	24.06 (± 3.75)	23.32(± 2.57)
**Duration of RG (months)**	–	167.92 (± 102)

All participants had direct sunlight exposure of less than 1 hour per day. The RG group had more than 4 servings/day of plant protein (the control group had 3 or fewer servings). The FFQ categories are described in the “Methods” section

**Table 2:** Participants’ blood biochemistry data

Groups	Control (***N*** = 64)	RG (***N*** = 56)	***P***-value
**TIBC**	339.97 (± 47.63)	337.3(± 45.40)	0.69
**Fe**	88.16 (± 37.57)	78.17 (± 32.46)	0.20
**K**	4.30(± 0.35)	4.25 (± 0.39)	0.54
**Na**	141.5 (± 1.78)	141.38 (± 1.73)	0.77
**Cl**	105.07(± 1.98)	106.26(± 2.35)	0.06
**Ca**	2.26 (± 0.11)	2.22 (± 0.15)	0.18

The unit of total iron-binding capacity and Fe is μg/dL. The unit of K, Na, Cl, and Ca levels is mmol/L. The RG group had more than 4 servings/day of plant protein (the control group had 3 or fewer servings). The FFQ categories are described in the “Methods” section. All values are represented as the mean ± SD. All data were analyzed using SPSS 18.0. Statistical analysis was performed using one-way ANOVA. A *p* value less than 0.05 was considered statistically significant. *p < 0.05, **p < 0.01

**Fig. 1:**
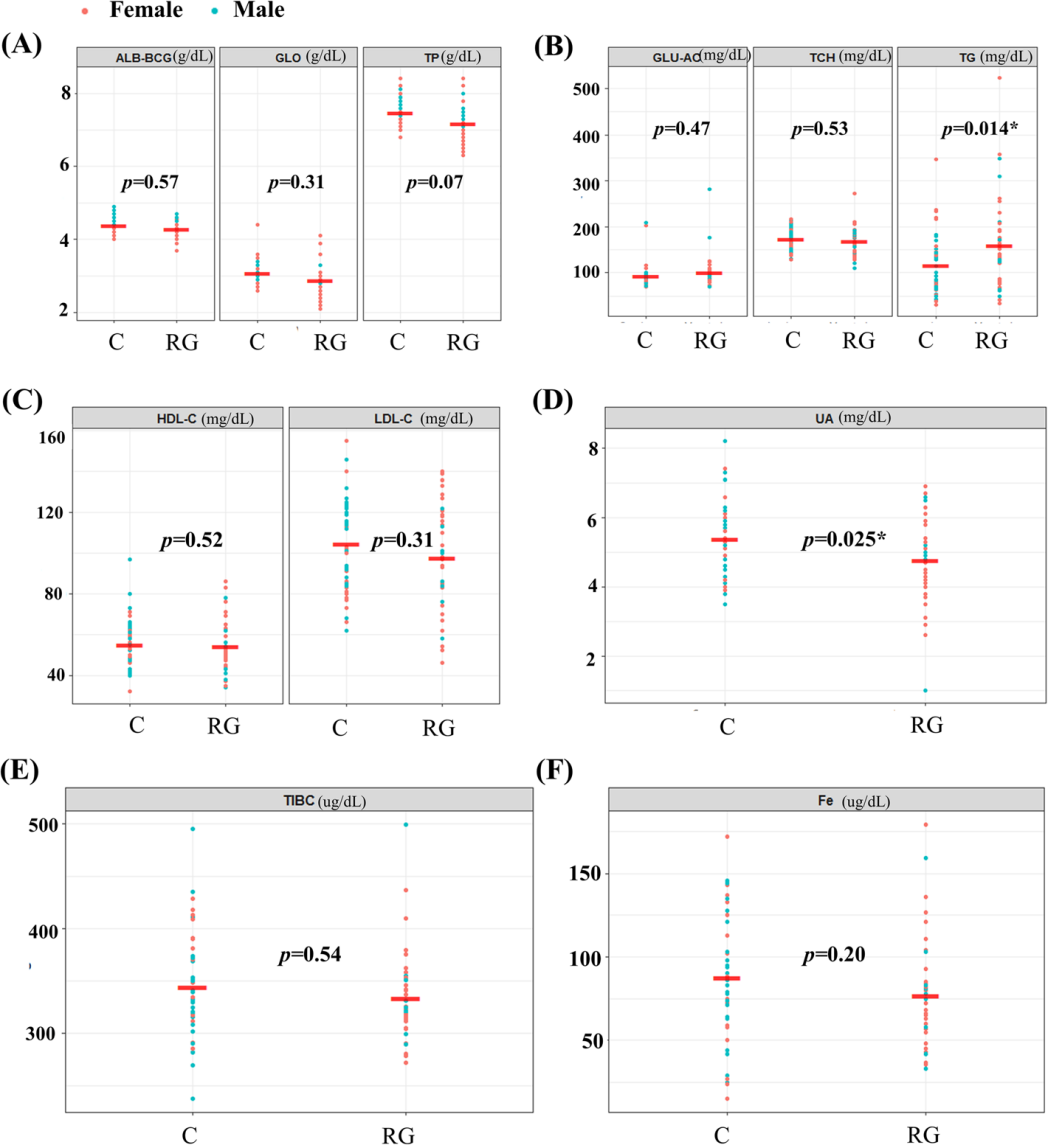
Biochemical characteristics of the blood samples of 120 participants. Volunteers were divided into the RG group and control (C) group. All values are presented as mean ± standard deviation (SD). The parametric *t* test and nonparametric Mann–Whitney *U* test for independent samples were used to compare groups. A *p* value of less than 0.05 was considered to indicate statistical significance. *p < 0.05, **p < 0.01. ALB: albumin; BCG: bromocresol green method, GLO: globulin, TP: total protein, GLU-AC: glucose-AC, TCH: total cholesterol, HDL-C: high-density lipoprotein cholesterol, LDL-C: low-density lipoprotein cholesterol, BUN (blood urea nitrogen). Red dots: female participants, green dots: male participants

The baseline characteristics of the 2 groups were determined through blood biochemical tests, and a sex-based division was applied (male: blue dots, female: red dots) (Fig. 1). Compared with the control group, the RG group exhibited lower serum uric acid levels (5.34 ± 1.13 mg/dL vs. 4.75 ± 1.21 mg/dL; *p* = 0.023)(Fig. 1D). Moreover, triglyceride (TG) levels were significantly higher in the RG group compared with the control group (160.89 ± 50 mg/dL vs 114.84 ± 44.02 mg/dL; *p* = 0.014) (Fig. 1B). Other lipid metabolism–based parameters, such as low-density lipoprotein (LDL) and high-density lipoprotein (HDL) levels, were not significantly different between the 2 groups (Fig. 1C). The total iron-binding capacity and free iron concentration, which are correlated with iron storage and transport, were also not significantly different between the 2 groups (Fig. 1E). These results indicate that most of the serum components were evenly distributed, but the RG group exhibited significantly higher TG levels and significantly lower levels of blood urea nitrogen.

### Genistein affects skin pH and pigmentation in vivo

**Fig. 2:**
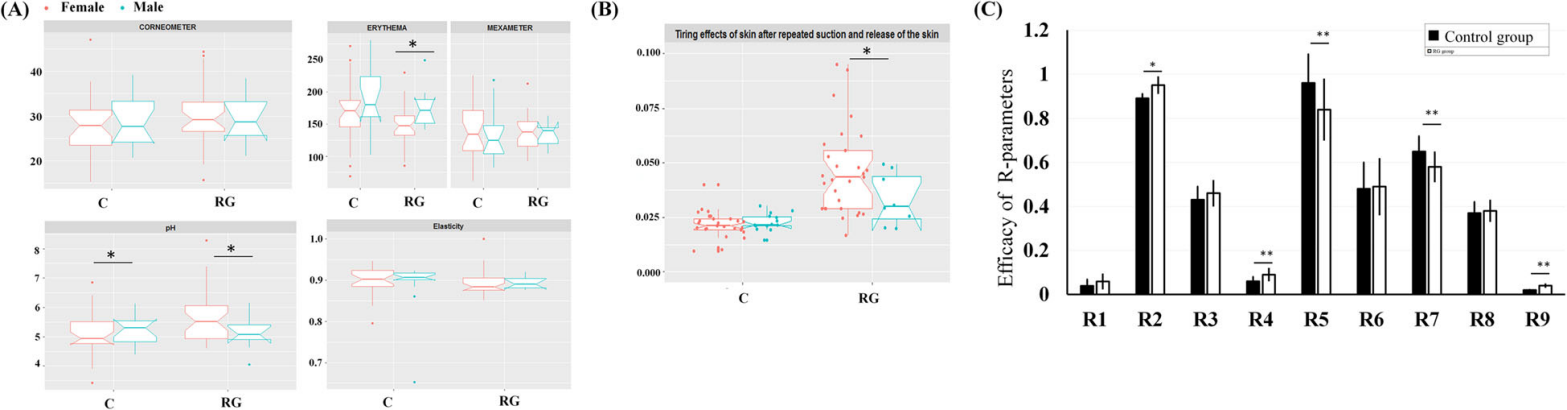
Participants’ skin parameters. (**A**) Skin hydration was assessed using a corneometer. Basic values for erythema and melanin were measured using a pigmentation probe (mexameter). A skin elastic meter was used to detect total skin elastic. All values are presented as mean ± SD. Participants were divided into the RG group and control (**C**) group. Red box: female participants, green box: male participants. All data were analyzed using SPSS version 18.0. A *p* value of less than 0.05 was considered to indicate statistical significance. *p < 0.05, **p < 0.01. (**B**) Advanced skin elasticity assay. Skin was subjected to constant negative pressure of 350 mbar in the time–strain mode for 18 s followed by relaxation for 2 s. (**C**) Elasticity parameters (R0–R9 and F0–F1) were calculated from the deformation curve and in accordance with the suction applied using the Cutometer MPA580. Bars represent the mean ± SD. (*N* = 5; each test was performed at least 5 times). R1 indicates skin total deformation; R2, skin gross elasticity; R3, skin amplitude; R4, last minute amplitude; R5, pure elasticity of the skin; R6, skin sagginess; R7, biological elasticity; R8, skin pliability; and R9, skin tiring effect. The black column represents the control group, and the white column represents the RG group. *p < 0.05; **p < 0.01, compared with other groups

To analyze the effect of genistein on human skin, different methods were used to analyze the various skin parameters (moisture, elasticity, and pigmentation) of the 2 groups (Table 3). We observed no significant differences between the 2 groups in terms of skin hydration, elasticity (skin extensibility), melanin, or the erythema index. Skin pH levels were significantly higher in the RG group than in the control group (5.51 ± 0.79 vs. 5.20 ± 0.67; *p* = 0.05). We further explored whether sex was an influencing factor. We grouped the individuals on the basis of sex and genistein intake and then analyzed the aforementioned skin parameters (Fig. 2A). In the RG group, men had a significantly higher erythema index than women (*p* = 0.04). Skin pH levels were higher among the women than among the men in the RG group. Conversely, skin pH levels were significantly higher among the men than among the women in the control group (*p* = 0.02).

**Table 3:** Participants’ skin parameters

Groups	Control	RG	*P-*value
Melanin index	146.58 (± 37.11)	140.21(± 30.15)	0.40
Erythema index	157.68 (± 44)	147.86 (± 32.15)	0.26
Hydration	28.18 (± 6.27)	29.91 (± 6.14)	0.21
Elasticity	1752.2 (± 367.6)	2122.9 (± 1245.8)	0.40
pH	5.20 (± 0.67)	5.51 (± 0.79)	0.05*

The elasticity parameter indicates skin extensibility. Skin parameters were assessed using the Corneometer CM580. The RG group had more than 4 servings/day of plant protein (the control group had 3 or fewer servings). The FFQ categories are described in the “Methods” section. All values are presented as the mean ± SD. Statistical analysis was performed using one-way ANOVA. A *p* value less than 0.05 was considered statistically significant. *p < 0.05, **p < 0.01

To understand whether genistein intake had any effect on the various skin parameters, advanced skin elasticity measurement was used to analyze the skin after repeated suction and release. Dynamic elasticity was higher in the RG group than in the control group, and women had significantly higher levels than men in the RG group (Fig. 2B, *p* = 0.01). These results indicate that compared with the RG group, the control group had lower skin pH and a higher erythema index. In addition, the RG group, especially women in this group, had superior skin elasticity to the control group.

### Genistein improves skin elasticity in vivo

We analyzed a single skin parameter to explore the effect of an RG diet on skin elastin. The R values represent the results at the end of different measurement cycles and are presented in Fig. 2C. Compared with the control group, the RG group had more pronounced improvements in R2 (*p* = 0.04), R4, R5, R7, and R9 values (*p* < 0.01). However, no significant differences were observed in R1, R6, and R8 between the 2 groups. Overall, we revealed that skin elasticity significantly improved in the RG group.

**Fig. 3:**
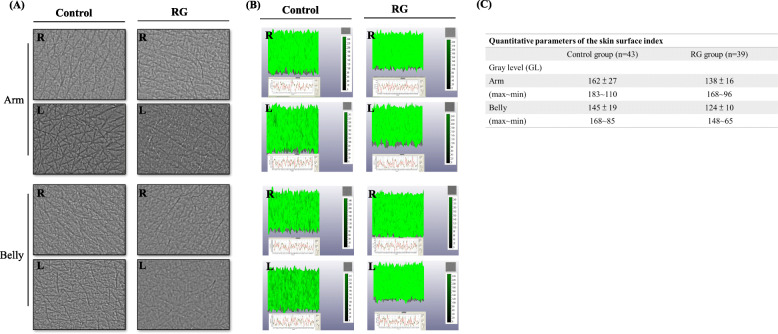
Skin textures and patterns. (**A**) Skin texture (arm and abdomen) and the distance between the highest peak and lowest valley. Skin appearance (magnification: 20×) from magnification device designed for observing human skin. R: right side, L: left side. (**B**) The gray level. (**C**) Quantitative parameters of the skin surface index [skin smoothness (Sesm), skin roughness (Ser), and wrinkles (Sew)] are shown underneath. The scale range (0–240) represents the depth of the skin. The GL can be obtained from the output data

A magnification device designed for observing human skin was used to observe the texture of the skin and the distance between the highest peak and lowest valley on the skin’s surface. The gray level (GL) was obtained (Fig. 3 A and B) and used to determine skin smoothness, roughness, and wrinkling. The abdomen and arm skin of the control group were thicker and had a denser population of skin cells compared with those of the RG group (arm: 162 ± 27 vs 138 ± 16, *p* = 0.053; abdomen: 145 ± 19 vs 124 ± 10, *p* = 0.06). The GL was obtained from detector the output data, as illustrated in Fig. 3C. The results confirmed that an RG diet could affect skin parameters; such a diet improved skin elasticity, reduced pigmentation, and maintained weak acidity, which protects the skin.

### Correlation between genistein intake and skin parameters in vivo

To understand the correlation between skin parameters and RG diet duration, we analyzed the RG diet duration (in months), pigmentation (melanin and erythema), skin hydration, and skin elasticity in the RG group and the control group. No skin parameter (melanin index, erythema index, or hydration) had a significant correlation with RG diet duration (*p* = 0.932, 0.446, and 0.077, respectively). When only the data of women in the RG group were analyzed, a positive correlation was discovered between skin hydration and RG diet duration (*p* = 0.044). Moreover, women in the RG group had comparison favorable skin hydration. The RG diet lasted 1 month, and the skin’s water retention was 0.014% higher after this diet (*β* = 0.014 ± 0.006, *p* = 0.044; Table 4). Individual analyses of the correlations between skin elasticity parameters and diet duration revealed that R5, R7, and R9 had significant positive correlations with RG diet duration. Using these results and the FFQ scores, we observed that upon the intake of protein derived from soybean, the participants had improved skin hydration. We divided the RG group into 3 groups: those with high, moderate, and low soybean intake (Table 5). The high-intake group had the best skin hydration (7.28 vs. 1.12 [moderate] and 0.01 [low], *p* = 0.007). These data indicate a positive correlation between RG diet duration, soybean intake, and skin hydration.

**Table 4:** Relationship between skin parameters and time

Characteristics	Time (months)		
	***β***	SEM	***P***-value
Melanin index	− 0.003	0.034	0.932
Erythema index	− 0.03	0.039	0.446
**Hydration**	0.011	0.006	0.077
Female	**0.014**	**0.006**	0.044*
**Elasticity**			
R2	0.741	0.690	0.574
R5	0.532	0.015	0.046*
R7	0.221	0.030	0.022*
R8	0.101	0.012	0.032*

Samples were analyzed to determine the relationships between rich genistein intake duration and skin parameters. Linear regression analysis was applied to investigate the relationship between the continuous variables and independent variable. A *p* value of less than 0.01 was considered statistically significant and labeled **. *β*: as the duration of a diet rich in genistein diet increased, changes were observed in this parameter. *SEM*, standard error of the mean. The skin parameters (namely melanin, erythema, hydration, and elasticity) were assessed using the Corneometer CM580

**Table 5:** Correlation between participants’ skin parameters and the FFQ results

Skin hydration	RG diet time (months)					
	Ave	SD	***β***	SEM	***n***	***P***-value
**Soybean product**						
**Frequency uptake**						
**1. High**	**34.13**	**7.22**	**7.28**	**2.61**	**16**	0.007**
**2. Moderate**	**27.97**	**5.56**	**1.12**	**2.31**	**55**	
**3. Rare**	**26.85**	**2.12**	**0.01**		**7**	

FFQ categories are described in the “Methods” section. Subjects who ate more than 4 servings/day were defined as having high intake; those who ate 2–3 servings/day were defined as having moderate intake; and those who ate fewer than 2 servings/day were defined as having low intake. ANOVA (analysis of variance) was used for statistical analysis. *Ave*, average, *SD*, standard deviation, *SEM*, standard error of the mean, *n*, number. A *p* value less than 0.01 was considered statistically significant and labeled **

### Genistein reduces skin cell inflammation caused by UVB irradiation in human keratinocytes

**Fig. 4:**
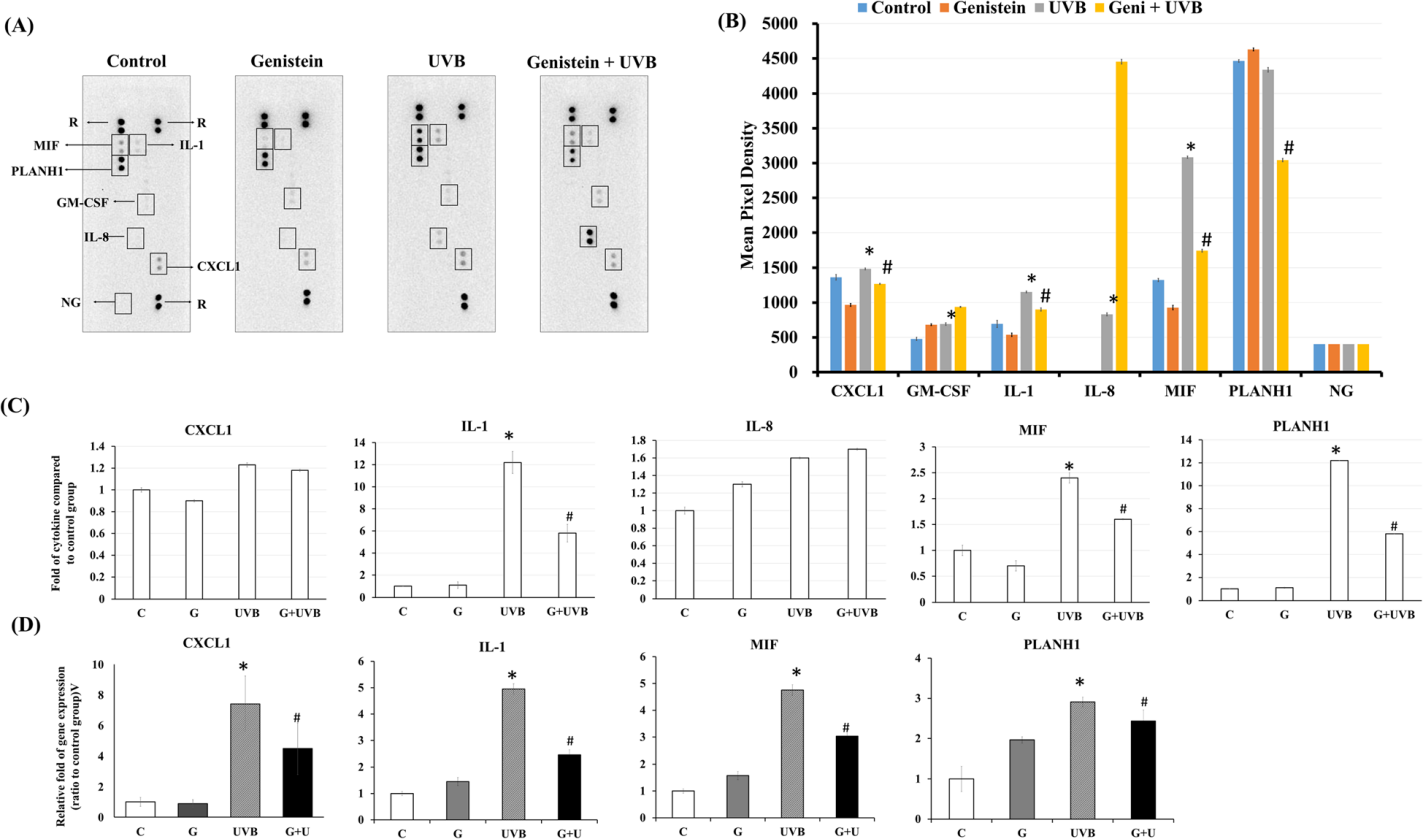
Effect of genistein on UVB-irradiated skin cells. (**A**) Cytokine array measurements were performed on cell culture supernatants harvested from cultures pretreated with genistein (5 μM) for 24 h with or without UVB (50 mJ/cm^2^) irradiation. Each blot represents the immunoreactive staining of respective antibodies. Staining is absent in the negative control and blank slots. R1, R2, and R3 were used to determine the accuracy of the procedure. NG, negative control group. (**B**) Cytokine levels were quantified and analyzed using image analysis software. The relative expression levels of cytokines were determined through a comparison of the pixel intensity of the respective blots relative to that of the positive control in the same array. The parametric *t* test for independent samples was used to determine differences between groups. Values are presented as the mean ± SD of 2 independent measurements. (**C**) Commercially available ELISA kits for analyzing the levels of CXCL1, IL-1, MIF, IL-8, and PLANH1 were used in accordance with manufacturer instructions to quantify fold changes. Relative cytokine release is expressed as a percentage compared with an untreated control. (**D**) The relative gene expression levels of cytokines were measured using Q-PCR. *p < 0.05; **p < 0.01, compared with other groups. ^#^*p* < 0.05 compared with the UVB group

To demonstrate that genistein reduced the degree of UVB-induced skin aging, human keratinocyte (HaCaT) cells were pretreated with genistein and then exposed to UVB irradiation. We confirmed the toxicity of genistein for HaCaT cells by performing an MTT assay, in which 5 μM was the treatment concentration selected (Supplementary Fig. 1). We analyzed the expression of proinflammatory cytokines by using a cytokine array; genistein pretreatment reduced the amounts of proinflammatory cytokines (CXCL1, IL-1, and MIF) released by UVB-treated keratinocytes (Fig. 4A). An analysis of pixel density from the aforementioned array was conducted, and of the analyzed proinflammatory cytokines, MIF expression was the most significantly decreased by genistein treatment (Fig. 4B, *p* = 0.015). To confirm the results of this study, an enzyme-linked immunosorbent assay (ELISA) was used to reveal the performance of genistein in suppressing cytokine release (Fig. 4 C). The cytokine release results for IL-1 and MIF were consistent with those from the cytokine array. The gene expression levels of these cytokines were also analyzed using quantitative polymerase chain reaction (PCR), and the same trend was discovered (Fig. 4 D). The levels of cytokines secreted in response to the UVB-irradiated culture medium were higher than those observed in the control medium. Similarly, genistein treatment released significantly lowered the levels of CXCL1, IL-1, and MIF compared with the UVB irradiation group (*p* < 0.05). These results suggest that decreases in IL-1 and MIF cytokine secretion were significantly genistein dependent.

### Topical application of genistein for reducing photoaging in an animal model

**Fig. 5:**
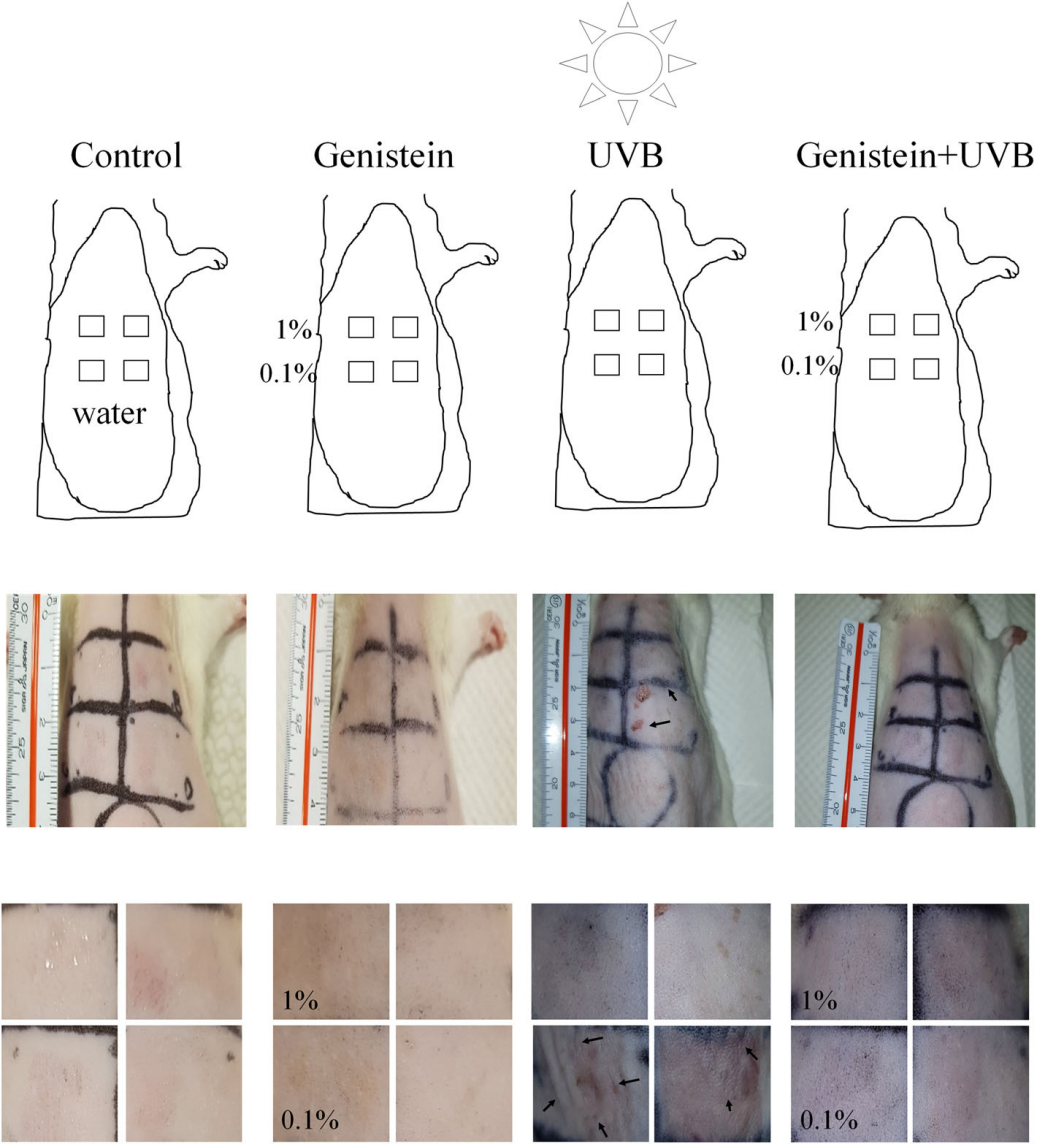
Animal model observations from the application of genistein. Male SD rats (3 to 4 weeks old) were divided into 4 groups (*n* = 6 per group). The hair on the back of the rat was removed 2 to 3 days before the experiments. The back of each animal was marked with India ink, and the marked area was divided into 4 squares (0.3 × 0.3 cm^2^ each); these squares were treated with 10 μL of each concentration (0.1% and 1%) of genistein solution in water, and one square was treated with water as a control area. The rats were pretreated with genistein once before they were exposed to UVB radiation (50 mJ/cm^2^); subsequently, skin wrinkling was investigated (magnification: 10×)

An animal model was used to determine whether the topical application of genistein affected UVB-induced skin aging. Two areas and duplicate areas on the dorsal skin of the same rats were marked and treated with 0.1% or 1% genistein. The genistein solution was applied to the dorsal skin of the rat for 24 h; the skin of the rats was then exposed to UVB light. The skin of the control and genistein groups (0.1% and 1%) did not change; however, the dorsal skin of the UVB-treated control group had prominent folds and wrinkles (black arrow), with tissue damage noted (Fig. 5).

## Discussion

This study examined the effects of genistein on UVB-induced skin aging and inflammation by using 3 models (in vivo, cell, and animal models). Our results demonstrated that genistein promoted skin hydration, decreased the amounts of UVB-induced proinflammatory cytokines released, and reduced the severity of UVB-induced wrinkles. We suggest that exposure to genistein may be beneficial for preventing light-induced skin aging.

In the cell model, among the 35 proinflammatory cytokines induced by UVB irradiation (Fig. 4), genistein significantly decreased the expression of CXCL1, IL-1, and MIF (*p* < 0.05); among them, genistein had the most significant inhibitory effect on MIF. MIF plays a critical role in the pathogenesis of UVB-induced nonmelanoma skin cancer [15]. MIF overexpression has been implicated in chronic inflammatory diseases and malignancies [16]. MIF deficiency significantly reduces acute inflammatory responses in the skin following UVB exposure [17]. We demonstrated that genistein effectively inhibited the expression of MIF. However, CXCL1, IL-1, and MIF cytokines did not differ in the blood samples of the two groups totaling 120 healthy participants. We speculate that MIF may be an indicator of skin cancer, meaning that it does not appear in large quantities in the blood of healthy people. However, the skin cell line used in this study was an immortalized model. Skin cell lines were well modeled to observe the regulatory influence of genistein on MIF. We suggest that genistein has potential use for skin cancer treatment.

Studies have shown that UVB irradiation causes skin injuries and oxidative damage through various pathways, such as the mitogen-activated protein kinase and nuclear factor-kappa beta (NF-κB)/p65 pathways [18, 19]. We also further proved that some proinflammatory cytokines (IL-6, IL-8, MCP-1, and COX-2) were stimulated by UVB irradiation through the NF-κB/p65 pathway in keratinocytes and a mouse model [1]. However, those cytokines were also analyzed in the cell model of the present study and found to be unaffected by genistein treatment. Therefore, we surmise that the effects of genistein were not related to the NF-κB/p65 pathway. Future experiments can explore the pathways through which genistein inhibits MIF expression in UVB-irradiated skin cells.

Through animal experiments (Fig. 4) and those involving human participants (Fig. 3), we confirmed that genistein significantly reduced inflammation and the severity of UVB-induced wrinkling. Genistein exhibits beneficial effects against skin aging through the increased synthesis of collagen and hyaluronic acid [20], the reduction of MMP expression, and the stimulation of fibroblast proliferation [21]. Collagen is synthesized primarily by fibroblasts residing within the dermis and is the cause of skin’s strength and elasticity, which are closely related to skin hydration and the formation of wrinkles. Our results demonstrated that genistein improved skin elasticity, reduced pigmentation, and maintained weak skin acidity. We must clarify the mechanism of genistein’s regulation of skin hydration through future investigations.

Studies have indicated that genistein has estrogen-like effects [22, 23] and has a higher affinity with estrogen receptors than other isoflavones have [24, 25]. Therefore, genistein’s effects are usually more pronounced in women. In our study, women had higher hydration and elasticity levels as well as lower LDL levels than the mean levels in the control and treatment groups (Fig. 1). Sex and hormones may explain these findings. However, the age range of the 120 participants was 54–56 years. In the RG group, the proportion of menopausal women was 44.7% (17 out of 38 women); in the control group, the proportion was 45.7% (16 out of 35 women). This result indicates that gender and menopausal status are just one of many influencing factors. One study indicated that treating postmenopausal skin with estrogen achieved superior results when isoflavones were used [22]. This result is not consistent with our results; more female participants are required to confirm whether hormones affect skin parameters.

Genistein has been tested in cosmetic antiaging preparations, and it has achieved notable results in terms of skin elasticity improvement, antiphotoaging, and skin cancer prevention. Cosmetic creams containing genistein have been used to alleviate skin dryness and wrinkles [26]. However, few studies have explored the effects of long-term genistein application on the physiological condition or pH of the skin. The RG diet duration (the time required for a genistein-rich diet to improve health) in the present study was long (average: 168 ± 102 months); thus, our data can be used to observe the relationship between genistein intake and duration. Our data demonstrated that the skin pH levels of all participants were within the normal range, although they were higher in the RG group than in the control group. One study demonstrated that women have lower skin pH than men [27]. However, we posit that genistein intake can reduce the quantity of acid deposits on the skin and higher the skin’s pH. Animal protein is metabolized into lactic acid and secreted by the skin and through sweat. A study indicated that nutrient metabolism may affect the skin through the microbiota [28]. Dietary intake of fiber and polysaccharides can reduce the accumulation of acidic substances through changes to probiotics in the gastrointestinal tract, reducing the severity of skin acne and hair follicle inflammation [29]. Whether an RG diet regulates lactobacillus and then affects the skin’s pH is of interest. Many studies assessing nutrients and skin inflammation have been performed using an atopic dermatitis model [30, 31]. Few related studies have used healthy human skin because many variables can affect the results. In this study, we used various methods to demonstrate that high genistein intake was beneficial in both in vivo and in vitro models.

## Conclusion

Genistein provided protection against UVB-induced inflammation. We demonstrated that an RG diet could affect the skin condition of participants; their skin exhibited favorable elasticity and hydration. Genistein suppressed the UVB-induced expression of proinflammatory cytokines (CXCL1, IL-1, and MIF) in skin keratinocytes. Topically applied genistein effectively reduced the appearance of wrinkles caused by sun exposure in an animal model. The experimental process and results are presented in a graphical abstract. Genistein is a safe and natural compound that could be employed within novel anti-inflammatory agents for topical application.

## Methods

### Reagents and chemicals

Genistein and phosphate-buffered saline were purchased from Sigma-Aldrich (St. Louis, MO, USA). Dulbecco’s modified Eagle’s medium (DMEM), penicillin/streptomycin, and fetal bovine serum were purchased from Gibco-Invitrogen (Carlsbad, CA, USA). The Cutometer MPA580 (C+K, Köln, Germany) skin detector was provided by the Department of Dermatology of Hualien Tzu Chi Hospital (Buddhist Tzu Chi Medical Foundation, Taiwan). ELISA kits for CXCL1 (cat. DGR00B), IL-1β (cat. DLB50), MIF (cat. SMF00B), and Serpin E1/PAI-1 (cat. DSE100) were obtained from R&D Systems, Inc. (Minneapolis, MN, USA). The Human IL-8/CXCL8 Quantikine ELISA Kit (S8000C) was obtained from BD OptEIA Set and used in accordance with the manufacturer’s instructions (Becton Dickinson, NY, USA).

### Study design and participants

A total of 137 participants (17 with incomplete information) were recruited between August 2016 and January 2018 for a 2-year study conducted by the Department of Dermatology, Hualien Tzu Chi Hospital, Taiwan. Informed consent was obtained from all participants, and institutional review board (IRB) approval was obtained prior to study commencement (IRB 104-44-A, Department of Dermatology, Hualien Tzu Chi Hospital, Buddhist Tzu Chi Medical Foundation). All participants were assessed using the FFQ [32–35]. The widely used instrument has correlation coefficients between 0.29 and 0.47 [36]. The inclusion criteria were as follows: healthy individuals aged 31–80 years (to confirm the effect of a long-term RG diet on the skin, the participants had to be older than 30 years) with BMI < 35 kg/m^2^. The exclusion criteria were as follows: history of skin cancer, photosensitivity disorder or atopy, sunbathing history or use of sunbeds in the preceding 3 months, photoactive medication administration, pregnancy, smoking status, alcohol use disorder, and substance use disorder. Relevant information—average daily exposure to strong sunlight, alcohol consumption habits (e.g., frequency, type, and quantity of alcoholic drinks consumed), dietary intake assessment results, and supplementary intake (vitamin pills, etc.)—was acquired. The FFQ covered 23 food items, and the frequency categories were 4–6 servings/day, less than 2 servings/day, 1–3 servings/week, and almost never [14, 36]. The RG group had more than 4 servings/day of plant protein, the control group had 3 or fewer servings. To analyze the relationship between genistein exposure and skin parameters, those in the RG group were divided into 3 subgroups on the basis of their soybean intake (high group: more than 4 servings/day; moderate group: 2–3 servings/day; low group: less than 2 servings/day). The volunteers were asked to fast for 8 h before blood samples were drawn for biochemistry measurements. Analyses of TG, HDL, LDL, fasting glucose, and creatinine levels as well as general biochemical analyses were performed at the Department of Laboratory Medicine, Hualien Tzu Chi Hospital, Taiwan. Plasma, serum, and urine samples were taken, and skin images were captured. The morphological characterization of skin samples was conducted after optimized images were captured using a digital microscope (Leica ATC 2000) fitted with a camera (40× resolution).

### Skin parameter assay

Skin hydration was assessed using the Corneometer CM580. The detectors of MPA580 included a corneometer, pH meter, mexameter, and elasticity probes. Additional parameters that could be assessed using the skin elasticity probe included *U* and *R* values. A measuring capacitor shows changes in capacitance in accordance with the moisture content of a sample [37]. The mechanical properties of the epidermis samples were determined using a noninvasive suction-based skin elastic meter equipped with a 2-mm measuring probe (Supplementary Fig. 2). Interference from sunlight and cosmetic lotion was avoided. Participants’ forearm areas and abdominal area covered by clothing were selected for testing, and 3-cm × 3-cm regions were marked on both forearms and the belly (right and left sides). The skin was marked as the basis of the aperture of the probe at a constant negative pressure of 350 mbar in the time–strain mode for 18 s, followed by relaxation for 2 s; the procedure was performed twice. Various *R* values could be obtained to clarify the operation. We explain these parameters on the basis of two studies in which this technique has been applied [38, 39]. The R parameters provided by the cutometer are R2 (gross elasticity or overall elasticity of the skin), R5 (net elasticity), R7 (biological elasticity; the ratio of elastic recovery to total deformation), and R8 (pliability; the ability of the skin to return to its original state). The skin deformation curves generated by the cutometer were mathematically and statistically analyzed. Basic values for erythema and melanin were obtained using a pigmentation probe (mexameter). All readings were acquired at 25 ± 2 °C and 40 to 50% relative humidity.

### Animal model, cell culture, and drug treatments

Animals were purchased from the National Laboratory Animal Center and housed at the Laboratory Animal Center, Chung-Shan University. The animal feeding approach was that used in our previous study [1]. We determined the effects of the topical application of aqueous genistein solutions (0.1 and 1%) on skin parameters. The back of each animal was marked with India ink, and the marked area was divided into 4 squares. Each group contained 6 animals. All animal experimental protocols were approved by the Ethics Committee of Chung-Shan Medical University Experimental Animal Center (Institutional Animal Care and Use Committee [IACUC] approval number: 1837). For the cell culture model, HaCaT cells were grown in DMEM at 37 °C in a humidified incubator with a 5% CO_2_ atmosphere. Cells were seeded in 6-well plates at a density of 2 × 10^5^ cells per well and cultured for 24 h in DMEM with genistein (5 μM). The supernatant was collected and evaluated using a cytokine array.

### UVB exposure

UVB light was supplied using a closely spaced array of KLBiotech STS-1 sunlamps, and the protocol was the same as that in our previous study [1]. The output energy of the UVB (290–320 nm) light was measured using a UVB photometer (LT Lutron, UV-340A photometer, International Light, Taiwan). The energy output was 1.5 mW/cm^2^ and corrected using the Dermaray UV meter and detector (Gigahertz-Optik, Pochheim, Germany). All procedures were conducted in accordance with relevant guidelines and regulations for both human and animal studies.

### Reverse transcription PCR, quantitative real-time PCR, and cytokine assay

Total RNA was isolated using Trizol reagent (Life Technologies, Grand Island, NY, USA) in accordance with the manufacturer’s instructions. Reverse transcription PCR and quantitative real-time PCR (qRT-PCR were performed as previously described [1]. The primers for Q-PCR amplification are presented in Supplementary Table 1. Cell-conditioned media were centrifuged for 5 min at 1200×*g*, and supernatants were collected before evaluation of cell cytokine secretion profiles. The levels of IL-8 in the cell supernatants were measured using a BD Opt EIA Set Human IL-8 ELISA kit following the manufacturer’s instructions (Becton Dickinson). Levels of other cytokines (CXCL1, IL-1, MIF, and PLANH1) were determined using an ELISA kit.

### Statistical analysis

The measured values obtained for various parameters were analyzed using SPSS version 18.0. The parametric *t* test for independent samples was used to compare groups. Analysis of variance (ANOVA) was used for comparisons of more than 2 groups. A *p* value of < 0.05 was considered statistically significant.

## Supplementary Information

**Additional file 1: Supplementary Fig. 1.** Cell viability following treatment with various concentrations of genistein for the indicated time periods (24 and 48 h). An MTT assay was used to determine the effect of genistein treatment on HaCaT cell viability. Data are presented as mean ± SD from 3 experiments (*n* = 6). * *p* < 0.05; ** *p* < 0.01.

**Additional file 2: Supplementary Fig. 2.** Cutometer MPA580 measurements of the elasticity, hydration, pigmentation, and pH levels of the skin of participants’ upper limbs. Skin hydration was assessed using the Corneometer CM580. The detectors of the Corneometer CM580 included a corneometer, pH meter, mexameter, and elasticity probes. Participants’ upper and inner arm areas and abdomen area were selected for testing.

**Additional file 3: Supplementary Table 1.** Primer sequences for cDNA amplification of selected human genes.

## Data Availability

The data sets used or analyzed are available from the corresponding author upon reasonable request.

## Abbreviations

AST: Aspartate transaminase
ALT: Alanine transaminase
BMI: Body mass index
BUN: Blood urea nitrogen
ER alpha: Estrogen receptor alpha
FBS: Fetal bovine serum
FFQ: Food frequency questionnaire
MMP: Metalloproteinase
NSAIDs: Nonsteroidal anti-inflammatory drugs
PUFAs: Polyunsaturated fatty acids
Real-time PCR: Real-time polymerase chain reaction
ROS: Reactive oxygen species
TG: Triglyceride
TIBC: Total iron-binding capacity
UVB: Ultraviolet B
