# Supplementary figures and images for: Genistein protects against ultraviolet B–induced wrinkling and photoinflammation in in vitro and in vivo models
Source: Genes Nutr. 2022 Feb 24;17:4. doi: 10.1186/s12263-022-00706-x (PMC8903702; doi:10.1186/s12263-022-00706-x)

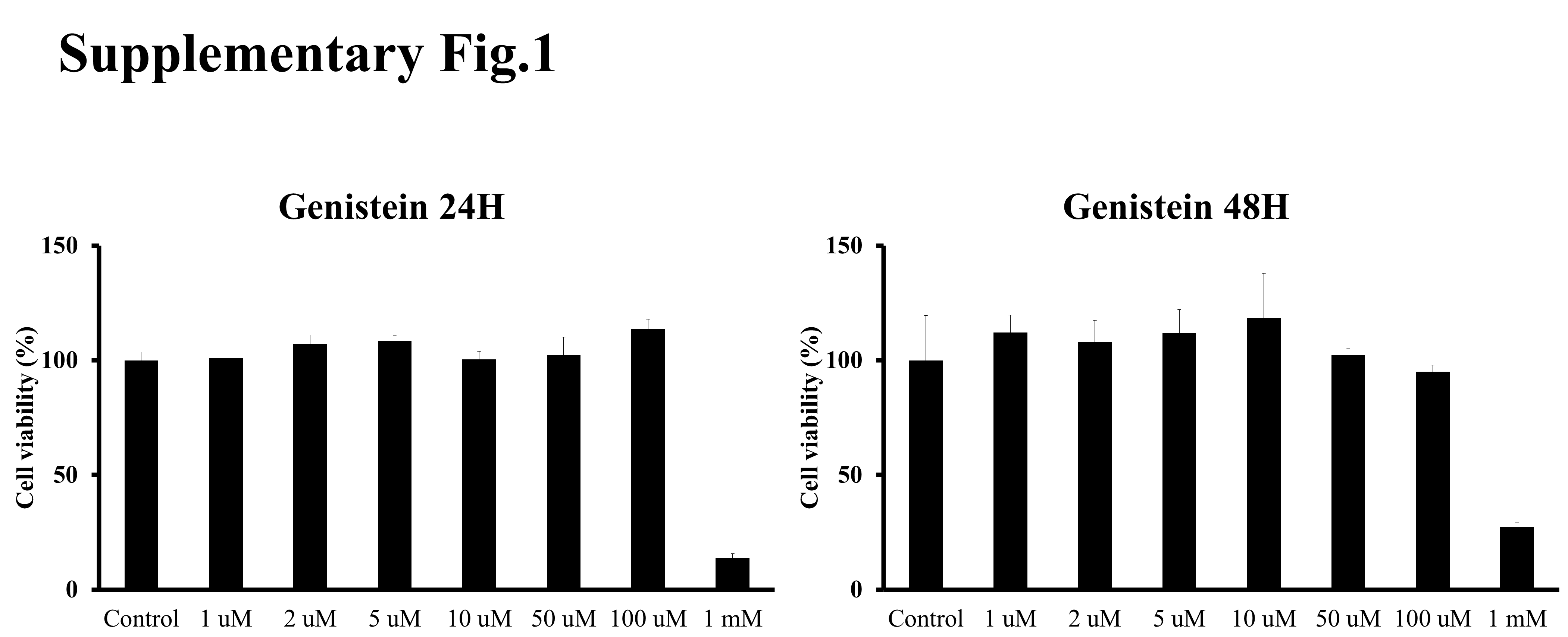

Supplement: Supplementary file 1 — Additional file 1: Supplementary Fig. 1. Cell viability following treatment with various concentrations of genistein for the indicated time periods (24 and 48 h). An MTT assay was used to determine the effect of genistein treatment on HaCaT cell viability. Data are presented as mean ± SD from 3 experiments (n = 6). * p < 0.05; ** p < 0.01. [file 12263_2022_706_MOESM1_ESM.png]

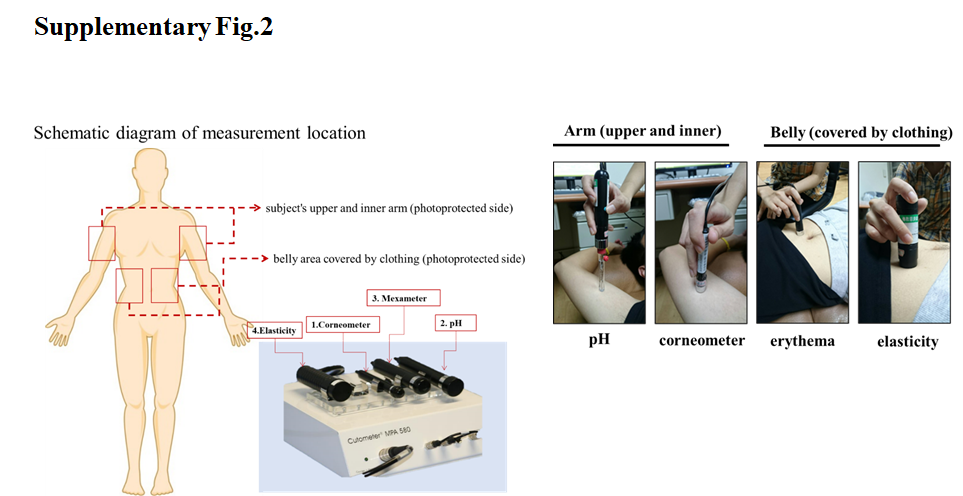

Supplement: Supplementary file 2 — Additional file 2: Supplementary Fig. 2. Cutometer MPA580 measurements of the elasticity, hydration, pigmentation, and pH levels of the skin of participants’ upper limbs. Skin hydration was assessed using the Corneometer CM580. The detectors of the Corneometer CM580 included a corneometer, pH meter, mexameter, and elasticity probes. Participants’ upper and inner arm areas and abdomen area were selected for testing. [file 12263_2022_706_MOESM2_ESM.png]
