# Supplementary material for: Genistein protects against ultraviolet B–induced wrinkling and photoinflammation in in vitro and in vivo models
Source: Genes Nutr. 2022 Feb 24;17:4. doi: 10.1186/s12263-022-00706-x (PMC8903702; doi:10.1186/s12263-022-00706-x)
Supplement: Supplementary file 3 — Additional file 3: Supplementary Table 1. Primer sequences for cDNA amplification of selected human genes. [file 12263_2022_706_MOESM3_ESM.docx]

**Supplementary Table 1. Primer sequences for cDNA amplification of selected human genes**

| Gene | Accession number | Primer sequences 5’🡪3’ | Position |
| --- | --- | --- | --- |
| GAPDH-F | NM_001357943.2 | ACCCACTCCTCCACCTTTGA | 890-909 |
| GAPDH-R |  | CTGTTGCTGTAGCCGAATTCAT | 990-969 |
| IL1A-F | NM_000575.5 | GTGCCGTGAGTTTCCCAG | 748-731 |
| IL1A -R |  | ACTCAATTGTATGTGACTGCCCA | 624-646 |
| CXCL1-F | NM_001511.4 | CGGAAAGCTTGCCTCAATCCT | 322-342 |
| CXCL1-R |  | GTCAGTTGGATTTGTCACTGTTC | 103-381 |
| MIF-F | NM_002415.2 | ATGTTCATCGTAAACACCAACGTGC | 99-123 |
| MIF-R |  | AGCTCGGAGAGGAACCCGT | 160-142 |
| SERPINE1-F | NM_001018068.2 | CTGTCTCAATGAACAATGGGTCAA | 3109-3086 |
| SERPINE1-R |  | TGCACTGGTGCTAATGTCTAAT | 3004-3025 |

The expression levels of mRNA in cell were normalized to those of glyceraldehyde-3-phosphate dehydrogenase.
